# Public health critical race praxis at the intersection of traffic stops and injury epidemiology

**DOI:** 10.1186/s40621-022-00375-9

**Published:** 2022-03-21

**Authors:** Michael Dolan Fliss, Frank R. Baumgartner, Paul Delamater, Steve W. Marshall, Charles Poole, Whitney Robinson

**Affiliations:** 1 Injury Prevention Research Center, University of North Carolina, CVS Plaza, Suite 500 CB# 7505, 137 E Franklin St,, Chapel Hill, NC 27599 USA; 2 Department of Political Science, University of North Carolina, 235 E Cameron Ave, Chapel Hill, NC 27514 USA; 3 Department of Geography, University of North Carolina, Carolina Hall, CB# 3220, Chapel Hill, NC 27599 USA; 4 Carolina Population Center, University of North Carolina, 123 W Franklin St, Chapel Hill, NC 27516 USA; 5 Department of Epidemiology, University of North Carolina, 170 Rosenau Hall, CB #7400 | 135 Dauer Drive, Chapel Hill, NC 27599 USA; 6 Injury Prevention Research Center, University of North Carolina, Chapel Hill, NC USA; 7 Department of Obstetrics and Gynecology, Duke University, 40 Duke Medicine Circle Clinic 1J, Durham, NC 27710 USA

**Keywords:** Traffic stop, Motor vehicle crash, Disparity, Crime, Law enforcement, Policing, Race, Anti-racism, Public health critical race praxis

## Abstract

**Background:**

Law enforcement traffic stops are one of the most common entryways to the US justice system. Conventional frameworks suggest traffic stops promote public safety by reducing dangerous driving practices and non-vehicular crime with little to no collateral damage to individuals and communities. Critical frameworks interrogate these assumptions, identifying significant individual and community harms that disparately impact Black, Indigenous, and People of Color (BIPOC) and low-income communities.

**Methods:**

The Public Health Critical Race Praxis (PHCRP) and multi-level frameworks from community anti-racist training were combined into a structured diagram to guide intervention and research teams in contrasting conventional and critical perspectives on traffic stops. The diagram divides law enforcement and drivers/residents as two separate agent types that interact during traffic stops. These two agent types have different conventional and critical histories, priorities, and perspectives at multiple levels, including individual, interpersonal, institutional, and cultural levels. Conventional solutions (identifying explicitly racist officers, “meet-a-cop” programs, police interaction training for drivers) are born from conventional frameworks (rewarding crime prevention regardless of cost, the war on drugs saves lives, driver behavior perfectionism). While conventional perspectives focus on individual and interpersonal levels, critical perspectives more deeply acknowledge dynamics at institutional and cultural levels. Critical solutions may be hard to discover without critical frameworks, including that law enforcement creates measurable collateral damage and disparate social control effects; neighborhood patrol priorities can be set without community self-determination or accountability and may trump individual and interpersonal dynamics; and the war on drugs is highly racialized and disproportionally enforced through traffic stop programs.

**Conclusions:**

Traffic stop enforcement and crash prevention programs that do not deeply and critically consider these dynamics at multiple levels, not just law enforcement-driver interactions at the individual and interpersonal levels, may be at increased risk of propagating histories of BIPOC discrimination. In contrast, public health and transportation researchers and practitioners engaged in crash and injury prevention strategies that employ law enforcement should critically consider disparate history and impacts of law enforcement in BIPOC communities. PHCRP, anti-racism frameworks, and the included diagram may assist them in organizing critical thinking about research studies, interventions, and impacts.

## Background

Law enforcement traffic stops are one of the most common entryways to the US justice system. Conventional public health frameworks suggest traffic stops promote public safety by reducing dangerous driving practices while having little if any collateral damage to individuals and communities. Conventional law enforcement frameworks (Scalia 1996) see traffic stops as a legally valid reason to investigate any person viewed with suspicion, which may lead them to target individuals based on their intersectional identities rather than driving behaviors. Viewed critically through racialized history and context, traffic stops have clear harms at the individual, interpersonal, institutional, and cultural levels. These harms, especially those disparately affecting Black, Indigenous, and People of Color (BIPOC) and low-income communities, must be weighed against stated public health benefits.

Traffic safety interventions, including popular Vision Zero campaigns (Conner 2017), require a critical view of the role that traffic enforcement can play in public safety. There are proven interventions to reduce crashes and racial disparities in traffic stops, including one we evaluated previously in *Injury Epidemiology* (Fliss et al. 2020). Injury epidemiology has a long history of applying critical thinking frameworks such as the Haddon Matrix while conceiving and critiquing interventions. This commentary combines two frameworks, the Public Health Critical Race Praxis (PHRCP) and community anti-racism trainings, into a diagram that supports public health practitioners, researchers, and community groups to think critically about involving law enforcement in crash prevention.

## Main text

### What are critical race theory (CRT) and public health critical race praxis (PHCRP)?

Critical Race Theory (CRT) ‘defines the set of anti-racist tenets, modes of knowledge production, and strategies a group of legal scholars of color in the 1980s organized into a framework targeting the subtle and systemic ways racism currently operates above and beyond any overtly racist expressions’ (Ford and Airhihenbuwa 2018). CRT distinguishes itself from both colorblind approaches to racism, such as a feminism or class critique disconnected from intersectional race realities, and from civil rights approaches that seek redress for racism without changing underlying racist structures.

Ford and Airhihenbuwa called for CRT’s inclusion in public health in 2010 (Ford and Airhihenbuwa 2010) and again in 2018 (Ford and Airhihenbuwa 2018), promoting the PHCRP as a semi-structured framework to facilitate the integration of CRT into public health research disciplines, including epidemiology, that produce and interpret evidence used for intervention evaluation and policy promotion.

The PHCRP has four foci and eleven affiliated, interrelated principles. The four foci are (1) contemporary patterns of racial relations, (2) knowledge production, (3) conceptualization and measurement, and (4) action. The eleven principles that relate to one or more foci are: (1) race consciousness, (2) primacy of racialization, (3) race as a social construct, (4) gender as a social construct, (5) ordinariness of racism, (6) structural determinism, (7) social construction of knowledge, (8) critical approaches, (9) intersectionality, (10) disciplinary self-critique, and (11) voice.

We have not formally been formally trained in PHCRP (Ford and Airhihenbuwa 2018), so humbly offer our application as much to demonstrate its application to traffic stops as to advance our own understanding. We also draw on previous experience with community anti-racism training (through dismantling Racism Works and the Racial Equity Institute) based on frameworks from the People’s Institute for Survival and Beyond (PISB) (The People’s Institute 2019). These characteristics of institutions (Okun 2010) overlap with PHCRP in many ways. Contrasting the conventional view of racism as purely interpersonal, PISB-influenced trainings present racism as both hierarchical and nested (i.e., internalized racial superiority and inferiority, interpersonal interactions, institutional settings, and cultural context). This model mirrors multilevel (Diez 2002) frameworks used in social epidemiology (Gee 2008), traffic crash research (Adanu et al. 2017), geography (Kim and Subramanian 2016), and criminology (Jones and Pridemore 2018) settings.

Since its introduction, CRT and the PHCRP have been increasingly used to guide study design, interpretation, and suggest areas for future research. These applications are varied, including public park features in Latino immigrant neighborhoods (García et al. 2016), lead water contamination in Flint, MI (Muhammad et al. 2018), and law enforcement “justifiable” homicides of Black men (García et al. 2016).

### Applying CRT/PHCRP to traffic stops

We follow the example from Gilbert and Ray (2015) by contrasting a conventional interpretation with a PHCRP interpretation for each principle in a tabular format based on literature review, community practice, and known disparities (Table 1). Themes from the conventional and critical contrasts are combined into a single figure describing these nested, multi-level dynamics within the unique nexus of traffic stops.

**Table 1:** PHCRP versus conventional view of traffic stop frameworks

Principle^a^	Affiliated focus^a^	Definition^a^	Conventional approach	PHCRP approach
1. Race consciousness	All	Deep awareness of one’s racial position; awareness of racial stratification processes operating in colorblind contexts	"Color blind" traffic stop interactions based on "objective" measures of crime and universal application of law. Race and of officer, driver, and passengers are irrelevant, as are demographics of neighborhoods, agencies, and political representation. Ignores existing stratification by race (e.g., segregation, income disparities, power and representation disparities, infrastructure investments) that further feed traffic crash and stop disparities	Understand role of individual race identities in decision making and interactions, e.g., internalized superiority and inferiority in implicitly and explicitly biasing interpersonal interactions. Acknowledge highly discretionary application of law and disconnect from measures of public health impact. Understand organization and neighborhood-level identity and demographic dynamics. Acknowledge and act equitably (not objectively) given racially asymmetrical distribution of stratifications (e.g., segregation, income disparities, power and representation, infrastructure investments). Adopt actively anti-racism frameworks
2. Primacy of racialization	Contemporary racialization	The fundamental contribution of racial stratification to societal problems; the central focus of CRT scholarship on explaining racial phenomena	Framing racial disparities as negative collateral byproducts instead of primary consequences of policing. Defensiveness on accusations of racial bias in interpersonal actions or decision making or when challenged by disparities in outcomes (e.g., differences in stop, search, etc. rates)	Acknowledge primacy of racialized policing, especially war on drugs and modern-day treatment of epidemics and poverty. Center histories of White supremacist law setting and the primary effectiveness of racism as an organizing suppression strategy. Contrast conventional frameworks with CRT frameworks for building study designs and interpreting results
3. Race as a social construct	Contemporary racialization, conceptualization and measurement	Significance that derives from social, political, and historical forces	Race is only conceived as an immutable, self-identified, biological construct. Race is synonymous with phenotype. No discussion of place- and time-specific changing definitions of race, self- and other-ascription of racial identity. No discussion of strengths and limitations of categorizing diverse people's phenotypes, cultural and language experiences, self- and other-ascribed identities, ancestry, etc., in limited race-ethnicity boxes. No discussion of political forces (capitalism, White supremacy) that drive disparate treatment by race	Acknowledge nuanced dynamics in assessing race, including place-specific passing (e.g., as White non-Hispanic), self- or other-identification of race-ethnicity, and the changing social definitions of race categories. Describe the legal treatment and protection of race and disparities juxtaposed against polices to promote White supremacy explicitly and implicitly. Contextualize traffic stop programs in decades of racism in general and law enforcement racist policies in particular: e.g., historical and present-day racialized war on drugs, enforcement of land use decisions, social control and broken-window policing
4. Gender as a social construct	Contemporary norms of masculinity, conceptualization and measurement	Significance of gender constructions that derive from social, political, and historical forces	Ignores contemporary masculine culture norms of officers and agencies, presenting them as gender-less or gender-neutral. Ignores gender demographic dynamics in driving. Ignores the place-specific, localized construction of gender norms and demographic distributions through policy enforcement (e.g., arrest of Black men for non-violent crimes, specific driving distributions)	Names, interrogates, and may act on masculine cultures aspects of enforcement: lone wolf policing, hierarchies, officer resistance to community authority, independence, binary thinking, production and individual advancement over community relationships. Gender-specific analyses of both drivers and officers, with critical discussion of measurement. Place-level analyses that acknowledge localized gender cultures and intersection of gender and race
5. Ordinariness of racism	Contemporary racialization	Racism is embedded in the social fabric of society	Racism is framed as a rare event between individuals (e.g., officer and driver), instead of a multi-level, pervasive oppressive force through history that produces experiences at all levels, including micro-aggressions, explicit racial discrimination, implicit bias, institutional policies, cultural preferences, and local, state, and national policies	Racism and its products (including traffic stop disparities) are discussed not only as (common) events, but a pervasive system that disallows the possibility of neutral interactions or policy and demands an explicitly anti-racist approach. Focus pulls back from single opportunity for racism (e.g., individual officer bias) to multiple opportunities for individuals, agency policies, and other related content areas (e.g., driving, poverty, representation) that interact at the nexus of traffic stops
6. Structural determinism	Contemporary racialization	The fundamental role of macro-level forces in driving and sustaining inequities across time and contexts; the tendency of dominant group members and institutions to make decisions or take actions that preserve existing power hierarchies	Sole focus of disparities is behavioral: behavior of the officer (e.g., explicit or implicit bias) and behavior of the driver (whether any behavior of could remotely, under any law, be rationale for a stop). No treatment of macro-level forces like income disparities, historical and current community disinvestment, patrol priorities or distribution. Agency and officer denial of responsibility to any structural causes in lieu of a tunnel-vision focus on whether a very specific interaction, separated from its contexts, could be rationalized. Focus on the behavioral is framed as objective, colorblind application of law and policy, even history reveals they were not constructed objectively	Analysis of traffic stops expand beyond the immediate and behavioral to institutions (e.g., law enforcement agencies), accounting for other structural disparities and may include multi-level components. Acknowledgement of pervasiveness of structural determinism, acknowledges and moves past defensiveness to wider conception of collective responsibility (especially parts of oppression that are no one person's job, e.g., a racism not requiring racists). White dominant institutions and white people in particular pay particular attention to disparate and compounding impacts, not just localized intentions. Institutions are directly accountable to a broad diversity of other communities and institutions, given the interrelatedness of structural determinism
7. Social construction of knowledge	Knowledge production	The claim that established knowledge within a discipline can be reevaluated using antiracism modes of analysis	Data collected on traffic stop forms (including race-ethnicity and gender identifiers), associating driving data, law enforcement administrative data (e.g., court fines and fees, arrest data) are all treated as objective with known, external meanings. Little attention given to hidden dynamics or limitations data generation process. Conventional frameworks are treated uncritically as universal, immutable, and ahistorical, without an origin in time, place, people, or power	Quantitative data, qualitative data, and implicit and explicit frameworks that drive meaning are treated as if they have social origins and are socially mutable, especially through a power lens. This includes traffic stop questions like why as many traffic stops occur as they do, when did those efforts start, and how have they changed; what do traffic stops prevent, when did we come to believe this,what is and isn't measured and who decided that, and what evidence exists for it; and racism questions like what has race-ethnicity meant in the past or in different places, how does racism operate now, and how might anti-racist action operate here and now
8. Critical approaches	Knowledge production, action	A social psychological approach to develop a comprehensive understanding of how individual biases develop prejudice and discrimination in social interaction	Knowledge produced is done so uncritically, with little attention to origin, deeper meanings, flaws, or implications. No consideration of data, information, knowledge, or wisdom hierarchy and how knowledge does or does not spread to others or deepen over time. Narratives are simple and likely separated from any considerations of shared responsibility, historical meaning, or possibility of wrong-doing on part of officers or government—excepting perhaps "bad apples" that are (again, uncritically) known to be explicitly racist	Data, assumptions, knowledge, and actions are all examined critically, particularly with an anti-racist lens. Agencies and governments share responsibility for not just enforcing, but perpetuating racism. Anti-racist agencies continually look for places to take improved action or stop action entirely if damaging to marginalized groups. Critical voices from community members and outside agencies are not ostracized and "othered," but welcomed and integrated. Data not collected, not just data collected, are considered critically
9. Intersectionality	Conceptualization and measurement, action	The interlocking and multiplicative approach to co-occurring social categories (e.g., race and gender) and the social structures that maintain them	Failure to consider the interacting dynamics of racism alongside sexism, homophobia, and capitalism—e.g., implicit and explicit suggestions that race and racism operate the same for all people using or ascribed a certain identity / label). Failure to adopt a multilevel approach to addressing disparities—e.g., focusing exclusively on implicit bias training and behavioral interventions	Address white supremacist culture components alongside (toxic) masculinity cultures and other privileges and marginalized identities. Act from a multi-tiered approach when addressing disparities, considering not only personal, but institutional and cultural levels of actions, e.g., considering patrol patterns and neighboring agency practices. Integrate traffic stop program interventions alongside anti-racist public health interventions in other areas, such as overdose and mental health response
10. Disciplinary self-critique	Action	The systematic examination by members of a discipline of its conventions and impacts on the broader society	Critical voices in local government, public health, and law enforcement are suppressed in favor of a united front. Exceptional stories and counter examples are unwelcomed. History is generally ignored, especially any history that paints a discipline in a negative light (e.g., racist history of policing, public health, and local government social control)	Critical voices are esteemed, rewarded, and developed. Critical frameworks are included in required training and treated as a conveyable skillset, not a magic alignment. History of intentional and unintentional racism within the discipline are taught with a focus on anti-racist action and change
11. Voice	Knowledge production, action	Prioritizing the perspectives of marginalized persons; privileging the experiential knowledge of outsiders within	Law enforcement is the sole voice in determining programs and producing knowledge about those programs, perhaps with some minimal accountability to local government. Marginalized population experiences can be "swept under the rug" because they may be relatively few. White and middle-class experiences are taken as the overall norm, driving attention away from experiences of marginalized groups. Exceptional events are treated as necessary sacrifices to maintain otherwise effective traffic stop programs. Only law enforcement determines whether programs work, their efficiencies, and the benchmarks of success	The stories and experiences (individually and collectively) of people who are stopped are prioritized, particularly those who are most marginalized (people of color, justice involved populations, non-English speakers, etc.). These communities lead determinations of not only how analysis is done, but how stop programs operate. In short, individuals and communities self-determine how they want to be patrolled and policed, or at least co-design stop programs with local agencies. The voice of those who are injured (e.g., by traffic crashes, assaults, or injuries from the justice system) are held up

Columns marked with (^a^) are reprinted verbatim from Gilbert and Ray (2015)

### Nested, multi-level, dual-agent PHCRP framework for traffic stops

**Fig. 1:**
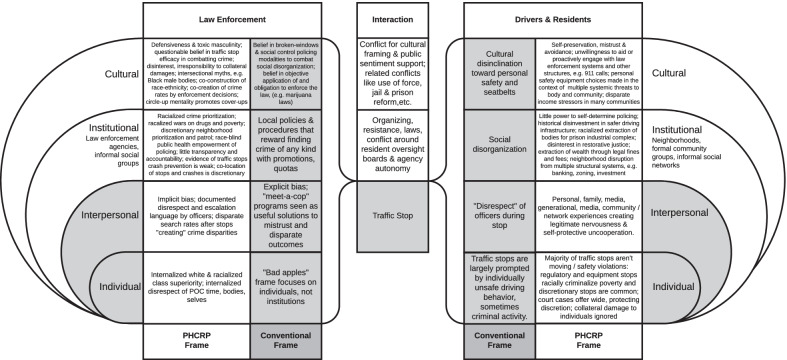
Nested, dual-agent PHCRP framework for critical examination of traffic stop dynamics and interventions. Conventional frameworks prioritize the individual (behaviors and internalized mindsets) and interpersonal levels, and limit interaction to focus on the traffic stop itself as a time and level of interaction. PHCRP emphasizes higher levels dynamics (institutional, cultural), root and historical causes, and collateral consequences

The conventional and PHCRP principles included in Table 1 could expand to its own full-length book. However, we believe there is utility in having a more condensed resource that connects the PHCRP framework to the traffic-stop setting visually. The PHCRP framework when expressed tabularly does not convey (1) the nested, multi-level dynamics of people, inter-personal interactions, institutions, and cultures, and (2) does not separate drivers and residents from law enforcement as unique loci for critical analysis, with their interaction being the nexus of the traffic stop. To that end, we built the following visual framework (Fig. 1) to contrast PHCRP and conventional frameworks nested within these multi-level structures, separating law enforcement and driver agents that meet at the traffic stop nexus. This figure and the PHCRP were used together to critically examine (1) limitations of a traffic stop intervention designed to reduce disparities and save lives (Fliss et al. 2020) and (2) issues with accurate measurement of traffic stop disparities (Fliss 2019).

## Conclusions

We recommend using PHCRP as a framework to guide more equitable and less unjust traffic stop policies and public health/law enforcement collaborations. We acknowledge PHCRP may be uncritically applied; its application is not automatic approval for interventions without leadership from disparately impacted communities. In contrast, a truly critical framework must contend with the possibility that few to no aspects of traffic stop programs may be equitable under the PHCRP. However, given training in PHCRP, communities, public health, and law enforcement may co-design traffic stop injury prevention programs that are tightly limited by anti-racist ethics, efficient and effective in application, and serve to deepen community trust instead of endangering it. Whether this is overly idealistic or can be done in practice is yet to be determined.

One way we have attempted to grapple with the application of PHCRP is to map it to dual-agent, nested traffic stop dynamics. While this diagram captures some nuances that the tabular form does not and may be useful as a blank template in group brainstorming sessions, it does not provide the same principle-specific contrasts as the full table. Researchers in other injury epidemiology settings may find utility in building diagrams that suit their research settings.

Both frameworks benefit from considering time in short (years) and longer (decades or centuries) scales. As acknowledged in the “the social construction of knowledge” principle, individual race-ethnicity is often tacitly used as a proxy for (or removed from) structural, historical, multi-generational racism and discrimination (VanderWeele and Robinson 2014). Frameworks such as PHCRP can help disentangle model variables by separating time and multi-level, structural phenomena such as racism. Sequencing important traffic stop related moments in time in the short term (e.g., pre-stop, stop, potential citation, potential search, potential arrest or use of force, etc.) and longer epochs (origins and historical milestones of policing and traffic stop enforcement) further separate those constructs.

We aspire to continual anti-racist learning and find PHCRP a useful framework for those efforts. Considering PHCRP principles under a multi-level framework of people, institutions, and culture may provide additional opportunities for reflection when applied to injury epidemiology related events such as traffic crashes and stops. We look forward to reading of its use in other injury epidemiology topics.

## Data Availability

Not applicable.

## Abbreviations

CRT: Critical race theory
BIPOC: Black, indigenous, and people of color
PHCRP: Public health critical race praxis
PISB: People’s Institute for Survival and Beyond
